# Lipoteichoic acids influence cell shape and bacterial division of *Streptococcus suis* serotype 2, but play a limited role in the pathogenesis of the infection

**DOI:** 10.1186/s13567-024-01287-w

**Published:** 2024-03-19

**Authors:** Servane Payen, Marie-Christine Giroux, Nicolas Gisch, Ursula Schombel, Nahuel Fittipaldi, Mariela Segura, Marcelo Gottschalk

**Affiliations:** 1 Research Group On Infectious Diseases in Production Animals (GREMIP) and Swine and Poultry Infectious Diseases Research Center (CRIPA), Department of Pathology and Microbiology, Faculty of Veterinary Medicine, University of Montreal, Saint-Hyacinthe, QC J2S 2M2 Canada; 2 Division of Bioanalytical Chemistry, Priority Area Infections, Research Center Borstel, Leibniz Lung Center, Borstel, Germany

**Keywords:** *Streptococcus suis*, lipoteichoic acids, cell defect, pathogenesis, cell adhesion and invasion, cytokines, virulence

## Abstract

*Streptococcus suis* serotype 2 is a major swine pathogen and a zoonotic agent, causing meningitis in both swine and humans, responsible for substantial economic losses to the swine industry worldwide. The pathogenesis of infection and the role of bacterial cell wall components in virulence have not been fully elucidated. Lipoproteins, peptidoglycan, as well as lipoteichoic acids (LTA) have all been proposed to contribute to virulence. In the present study, the role of the LTA in the pathogenesis of the infection was evaluated through the characterisation of a mutant of the *S. suis* serotype 2 strain P1/7 lacking the LtaS enzyme, which mediates the polymerization of the LTA poly-glycerolphosphate chain. The *ltaS* mutant was confirmed to completely lack LTA and displayed significant morphological defects. Although the bacterial growth of this mutant was not affected, further results showed that LTA is involved in maintaining *S. suis* bacterial fitness. However, its role in the pathogenesis of the infection appears limited. Indeed, LTA presence reduces self-agglutination, biofilm formation and even dendritic cell activation, which are important aspects of the pathogenesis of the infection caused by *S. suis*. In addition, it does not seem to play a critical role in virulence using a systemic mouse model of infection.

**Supplementary Information:**

The online version contains supplementary material available at 10.1186/s13567-024-01287-w.

## Introduction

*Streptococcus suis* is one of the most important bacterial pathogens of swine, causing significant economic losses to the swine industry. Moreover, it is a zoonotic agent representing serious risks for human health, especially in South-East Asian countries [1]. *S. suis* causes various diseases in pigs, with meningitis, arthritis and sudden death (septic shock) most frequently reported [2]. Among 29 described *S. suis* serotypes, the serotype 2 is the most commonly recovered from diseased pigs and human patients [3]. The pathogenesis of *S. suis* has not been completely elucidated. Although the role of some factors remains controversial, there is consensus that the virulence of the organism is complex and multifactorial [4]. The capsular polysaccharide plays a critical role in *S. suis* virulence, while cell wall components, such as proteins and lipoteichoic acids (LTA), have been proposed to contribute to the virulence of the organism [5–9].

LTA are macroamphiphiles that contain alditol- or glycosyl-phosphates as integral parts of their hydrophilic chain, which may have further modifications such as d-alanine or glycosyl residues [6]. They are anchored in the bacterial membrane by a terminal diacylglycerol unit and are major components of the cell-wall of most Gram-positive bacteria [5]. Whereas LTA are predominant in the subgroup of Gram-positive bacteria that contains DNA of a guanine and cytosine (G + C) content of < 50%, lipoglycans occur preferentially in the subgroup having a G + C-content of > 55%. Lipoglycans and LTA do not occur in parallel in the same organism and most likely replace each other functionally [10, 11]. Based on the architecture of repeating units, LTA are classified into five types. Type I contains polyglycerol phosphate, types II and III a glycosyl-glycerol-phosphate complex, type IV glycosyl-ribitol-phosphate and type V glycosyl-phosphate. Types II to V LTA are considered to be more complex [6], whereas type I LTAs are those most commonly found, among others, in *Staphylococcus aureus*, *Bacillus subtilis*, and *Listeria monocytogenes* [12–14]*.* On the other hand, *Streptococcus pneumoniae* contains a type IV LTA [15, 16]. It is important to mention that bacteria usually contain only one type of LTA [6]. Recently, the opportunistic pathogen *Streptococcus mitis*, like *S. pneumoniae* a member of the Mitis group of viridans streptococci, has been shown to contain two types of LTA. In addition to a type IV LTA very similar to that described in *S. pneumoniae*, a type I LTA was also observed [17]. For *S. suis* serotype 2 strains from different genetic and virulence backgrounds, an unexpected complexity of LTA molecules has been described. In addition to the type I LTA present in many other streptococci, a second mixed-type series of LTA molecules of higher complexity were found [7]. These second LTA molecules have similar constitutions as known from type II or type III LTA [6, 7]. Still, its role in the pathogenesis of the infection remains poorly understood [7].

LTA were over the years wrongly associated with Gram-positive bacteria septic shock, based on their apparent ability to activate leukocytes and stimulate an exacerbated production of pro-inflammatory mediators following recognition by TLR2 [18, 19]. However, recent studies have shown contamination of LTA extracts with lipoproteins and suggested that the latter were most likely the components recognized by the host cells and thus responsible for the inflammatory activation [7, 16, 20, 21]. Specifically in *S. suis*, lipoproteins have been shown to be ultimately the main activators of the innate immune system [7, 21–23]. Despite this limited contribution of LTA to the activation of the host immune response, d-alanylation of *S. suis* LTA has been shown to confer resistance to the action of cationic antimicrobial peptides and to the bactericidal effect of porcine neutrophils and murine dendritic cells. It also appears to modulate the bacterial association with blood leukocytes and to interfere in the deposition of complement on the bacterial surface [8, 9]. In addition, d-alanylation of LTA increases adhesion to and invasion of porcine endothelial cells [8] and could play a role in inflammasome regulation [9]. Notably, an isogenic *S. suis* mutant impaired in LTA d-alanylation was less virulent upon systemic infection in mice and pigs [8].

While the pro-inflammatory potency of LTA molecules might be relatively modest, their role in *S. suis* physiology and pathogenesis should not be underestimated [7]. To explore this, we generated a mutant strain of *S. suis* that entirely lacks LTA. This allowed us to investigate the true impact of LTA absence on bacterial fitness, adhesion and invasion capabilities in epithelial cells, survival in blood, the induction of inflammatory mediators in vitro, and overall virulence. Our results provide critical new insights into the nuanced role of LTA in the pathogenesis of the *S. suis* infection.

## Materials and methods

### Ethics statement

This study was carried out in accordance with the recommendations of the guidelines and policies of the Canadian Council on Animal Care and the principles set forth in the Guide for the Care and Use of Laboratory Animals. The protocols and procedures were approved by the Animal Welfare Committee of the University of Montreal (permit number Rech-1570).

### Bacterial strains and growth conditions

The bacterial strains and plasmids used in this study are listed in Table 1. The classical *S. suis* serotype 2 virulent European reference strain P1/7 was used as the wild-type throughout this study, including for construction of the isogenic mutants. When needed, a nonencapsulated *S. suis* serotype 2 mutant (Δ*cpsF*), previously produced in our laboratory was used as positive control [5]. *S. suis* strains were cultured in Todd Hewitt broth (THB; Becton Dickinson, Mississauga, ON, Canada) as previously described [24]. For in vitro cell culture assays, bacteria were prepared as previously described and resuspended in cell culture medium [5, 25]. When needed, antibiotics (Sigma-Aldrich, Oakville, ON, Canada) were added to the media at the following concentrations: for *S. suis*, spectinomycin (Spc) at 100 μg/mL; for *Escherichia* *coli*, kanamycin (Km) and spectinomycin at 50 μg/mL and ampicillin (Ap) at 100 μg/mL.

**Table 1:** **List of strains and plasmids used in this study**.

**Strain or plasmid**	**Characteristics**	**Reference**
*Streptococcus suis*		
P1/7	Virulent serotype 2 ST1 strain isolated from a case of pig meningitis in the United Kingdom	[26]
P1/7 Δ*ltaS*	Isogenic mutant derived from P1/7; in frame deletion of *ltaS* gene	This study
P1/7 Δ*cpsF*	Isogenic mutant derived from P1/7; in frame deletion of *cpsF*	[5]
*Escherichia coli*		
TOP10	F^−^ *mrcA* Δ(*mrr-hsd*RMS-*mcr*BC) φ80 *lacZ*ΔM15 Δ*lac*X74 *rec*A1 *ara*D139 Δ(*araleu*) 7697 *gal*U *gal*K *rps*L (Str^R^) *end*A1 *nup*G	Invitrogen
Plasmids		
pCR2.1	Ap^r^, Km^r^, pUC *ori*, *lac*ZΔM15	Invitrogen
pSET4s	Spc^r^, pUC *ori*, thermosensitive pG + host3 *ori*, *lac*ZΔM15	[27]

### DNA manipulations

Genomic DNA was extracted from the *S. suis* wild-type strain using InstaGene Matrix solution (BioRad Laboratories, Hercules, CA, USA). Mini preparations of recombinant plasmids were carried out using the QIAprep Spin Miniprep Kit (Qiagen, Valencia, CA, USA). Restriction enzymes and DNA-modifying enzymes (Fisher Scientific, Ottawa, ON, Canada) were used according to the manufacturer’s recommendations. Oligonucleotide primers (Table 2) were obtained from Integrated DNA Technologies (Coralville, IA, USA) and PCRs carried out with the iProof proofreading DNA polymerase (BioRad Laboratories, Mississauga, ON, Canada) or Taq DNA polymerase (Qiagen). Amplification products were purified using the QIAquick PCR Purification Kit (Qiagen) and sequenced using an ABI 310 Automated DNA Sequencer and ABI PRISM Dye Terminator Cycle Sequencing Kit (Applied Biosystems, Carlsbad, CA, USA).

**Table 2:** **List of oligonucleotide primers used in this study**.

**Name**	**Sequence (5′–3′)**	**Construct**
*ltaS*-ID1	AGATTCCCGTCATCAGTCC	p4Δ*ltaS*
*ltaS*-ID2	GATCATCGCTACGCTATAGAAG	p4Δ*ltaS*
*ltaS*-ID3	AAGTTTGTATAGCACAAACGGC	p4Δ*ltaS*
*ltaS*-ID4	GGTGAAGGTGGTCAACTTCAG	p4Δ*ltaS*
*ltaS*-ID5	ACCGTCATTCCACCAAAAC	p4Δ*ltaS*
*ltaS*-ID6	CCGTGAATTTGAACTGGTACTTCACAATAAAACCTCATGGTCC	p4Δ*ltaS*
*ltaS*-ID7	GGACCATGAGGTTTTATTGTGAAGTACCAGTTCAAATTCACGG	p4Δ*ltaS*
*ltaS*-ID8	GGTGATACTTGGTTGCCTG	p4Δ*ltaS*

### Construction of an LTA defective mutant

An isogenic Δ*ltaS* mutant was generated by precise in-frame deletion of the *ltaS* gene from the strain P1/7, by using splicing-by-overlap-extension PCRs as previously described [27, 28]. Briefly, the overlapping PCR deletion allele was cloned into plasmid pCR2.1 (Invitrogen, Burlington, ON, Canada), extracted with PstI and BamHI, recloned into the thermosensitive *E. coli-S. suis* shuttle plasmid pSET4sdigested with the same enzymes, giving rise to the knockout vector p4Δ*ltaS*. Electroporation of strain P1/7 and procedures to obtain the mutant were those previously described [27]. Allelic replacement was confirmed by PCR and DNA sequencing.

### Extraction of lipoteichoic acids and their analysis by native tris-tricine-PAGE

**Figure 1:**
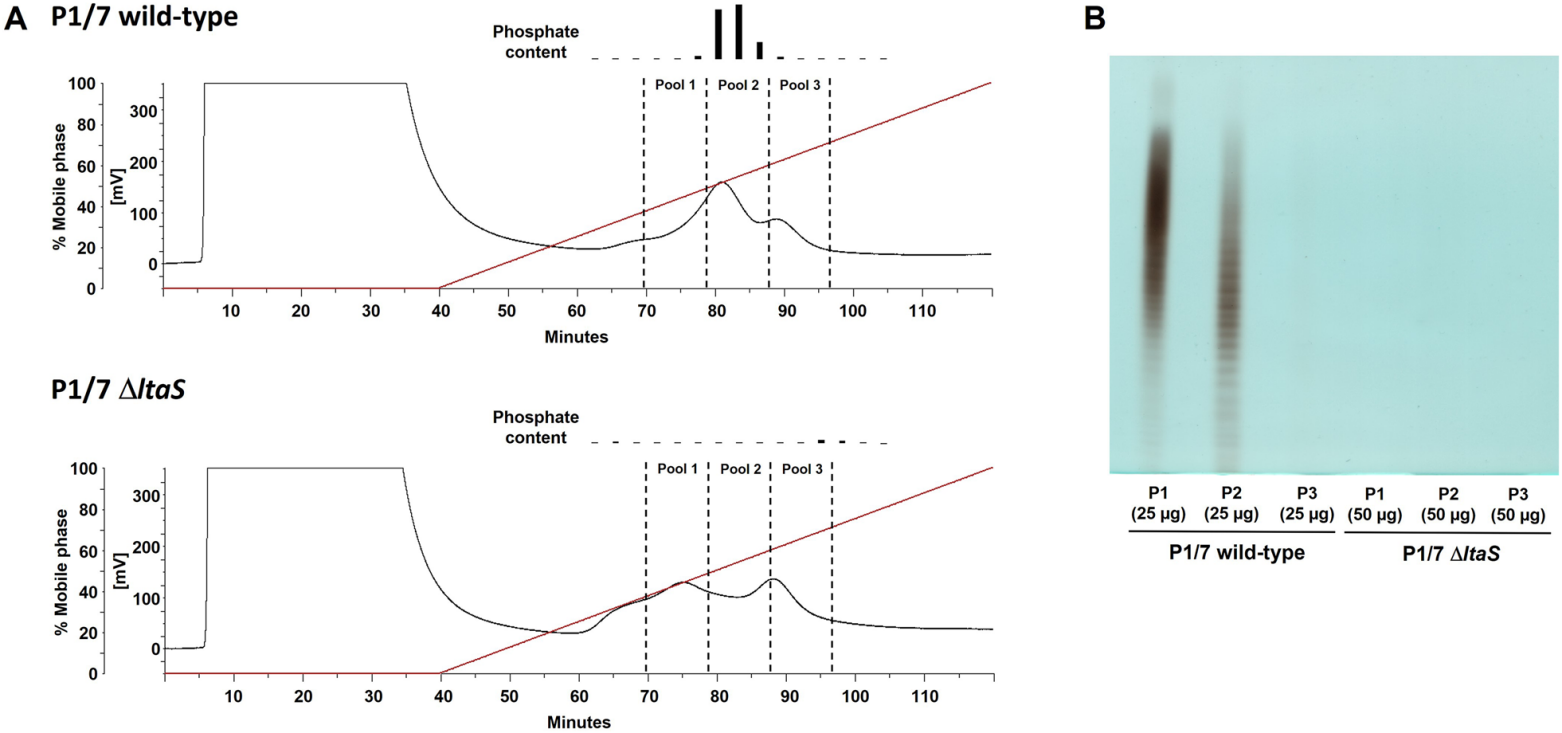
**LTA is absent in the *S. suis* serotype 2 P1/7 ∆*ltaS* mutant.** **A** Representative chromatograms (UV detection: λ = 254 nm) of HIC runs for LTA isolation from *S. suis* P1/7 wild-type (top) and ∆*ltaS* (bottom) including phosphate content. Indicated pools were subjected to (**B**) Tris-tricine-PAGE with combined alcian blue and silver staining. Applied material is the content of seven (wt) or five (∆*ltaS*) pooled runs, respectively [yields: P1: 2.12 mg, P2: 13.27 mg, 0.25 mg (wt); P1: 5.57 mg, P2: 2.79 mg, 0.69 mg (∆*ltaS*). Note: Phosphate content can slightly shift throughout individual HIC runs, so especially in pool 1 a partial LTA content is expectable.

LTA isolation and purification were performed as previously described [7]. Identification of LTA-containing fractions (3 min/fraction) in the purification by hydrophobic interaction chromatography (HIC) was achieved by a photometric phosphate test. LTA was mainly present in HIC fractions #27–29 (pool 2; see Figure 1A, top panel). In addition, the three fractions eluting before (pool 1, HIC fractions #24–26) and after (pool 3, HIC fractions #30–32) the LTA pool, respectively, were collected. Although displaying no significant phosphate content, the identical fractions of HIC runs performed with extracted material from the Δ*ltaS* mutant were collected (see Figure 1A, bottom panel). To visualize the presence or absence of LTA, all these fractions were subjected to native Tris-tricine-PAGE analysis essentially following a published protocol [29]. First, aliquots of the lyophilized pools were dissolved in Millipore-water in a concentration of 5 µg/µL. Portions of 50.8 or 70 µL (in dependency of the available sample amount, with 70 µL being the used default volume) were then subjected to benzonase and subsequent proteinase k digestion. For this, the respective portion was mixed with an equal volume of a mixture of Millipore-water/100 mM Tris–HCl (pH 8.0)/20 mM MgCl_2_/benzonase (25 U/µL) 0.8/1.0/0.5/0.2 (v/v/v/v). The 25 U/µL benzonase solution was freshly prepared by mixing the commercial 250 U/µL benzonase solution (1.01695.0001, Merck) with 100 mM Tris–HCl (pH 8.0)/20 mM MgCl_2_/Millipore-water in a 1:2:1:6 (v/v/v/v) ratio. After an incubation for 2 h at 37 °C, a proteinase k solution (20 mg/mL; AM2548, Ambion) was added in a volume equivalent to 1/32 of this mixture and the resulting mixture further incubated for 2 h at 50 °C. The final solutions of such enzymatic digests have a theoretical LTA concentration of 2.42 mg/mL. If samples were applied on the same day to the Tris-tricine PAGE, storage until use was at 4 °C, longer storage was at −20 °C. From HIC-pools of *S. suis* P1/7 wild-type 25 µg material (theoretical LTA) in 40 µL solution [prepared as follows: 12.9 µL enzymatic digest, 24.6 µL Millipore-water, 12.5 µL 4 × loading dye (according to [29])] were loaded on the PAGE, for HIC-pools of *S. suis* P1/7 Δ*ltaS* 50 µg material (theoretical LTA) in 40 µL solution [25.8 µL enzymatic digest, 11.7 µL Millipore-water, 12.5 µL 4 × loading dye] were used. Between each lane with sample one lane was filled just with 40 µL of 4 × loading dye/Millipore-water (1:3, v/v). Electrophoresis was performed at 14 mA (gel dimension: 16 cm × 14 cm × 0.75 mm) and 4 °C for 777 min in a Hoefer SE600 Gel Electrophoresis Unit (Hoefer Inc., Holliston, MA, USA). Subsequent sequential alcian blue [29] and silver staining [30] as performed as described, respectively.

### Bacterial surface hydrophobicity assay

Relative surface hydrophobicity of the *S. suis* wild-type, Δ*ltaS* and Δ*cpsF* mutant strains was determined by measuring adsorption to *n*-hexadecane as previously described [31].

### Self-aggregation and biofilm assays

For the self-aggregation assays, overnight cultures of *S. suis* were washed twice with phosphate-buffered saline (PBS), pH 7.3, and re-suspended in THB to obtain an optical density (OD) at 600 nm of 0.05. Samples were incubated at 37 °C for 24 h under static conditions and self-aggregation quantified as previously described [32, 33]. Biofilm formation capacity was determined as previously described [32] in the absence or presence of 5 mg/mL of porcine fibrinogen (Sigma-Aldrich).

### Electron microscopy

For scanning electron microscopy, fresh bacterial cells were cultured for 16 h in THB, and then placed on glass slides coated with poly-l-lysine (Sigma). Cells were fixed using 2.5% glutaraldehyde in 0.1 M cacodylate buffer for 1 h at 4 °C then rinsed 3 times in 0.2 M cacodylate wash buffer solution (pH 7.2). Post fixation was subsequently done using 1% osmium tetroxide (in 0.2 M cacodylate) before gradual dehydration through increasing ethanol concentrations (25, 50, 75, 95, and 100%). Carbon dioxide critical point drying (CPD) and gold sputtering were done on Leica EM CPD300 and Leica EM ACE600 instruments, respectively. The imaging was done using a Hitachi Regulas 8220 electron microscope with the SEM operation software Regulus 8200 series.

For transmission electron microscopy, fresh bacterial cells were cultured for 16 h in THB and were fixed by direct resuspension in 500 μL of 2.5% glutaraldehyde in 0.1 M cacodylate buffer and incubated for at least 1 h at 4 °C. Cells were then pelleted through centrifugation at 3000 × *g* for 3 min and washed 3 times in 500 μL 0.2 M cacodylate wash buffer solution (pH 7.2). 30–50 μL of wash solution containing bacterial cells was pipetted onto Formvar Carbon 200 mesh copper grids (Sigma-Aldrich) and negative staining done using 1% phosphotungstic acid (PTA) for 2 s before imaging at the INRS-CAFSB platform using a Hitachi H-7100 electron microscope with AMT Image Capture Engine (version 600.147).

### Antimicrobial peptide sensitivity

Antimicrobial peptide sensitivity assays were performed as previously described [8, 34]. Briefly, assays were carried out in sterile 96-well microtiter plates. The concentrations of logarithmic-phase *S. suis* cells were adjusted to approximately 10^4^ CFU/mL in 100 μL THB containing serial dilutions of one of the following antimicrobial compounds: colistin (0 to 200 μg/mL), polymyxin B (0 to 300 μg/mL), or Nisin (0 to 50 μg/mL). Plates were incubated for 24 h at 37 °C. The MIC was defined as the lowest antimicrobial concentration yielding no detectable bacterial growth as determined by measurement of the OD_600_.

### Bacterial adhesion and invasion assays to porcine tracheal epithelial cells

The neonatal porcine tracheal epithelial cell line (NPTr), frequently used in *S. suis* studies [35–37], was used. Cells were cultured until confluence as previously described [34], then infected with 1 × 10^6^ CFU/well (multiplicity of infection (MOI) = 10) of the different *S. suis* strains and incubated for 2 or 4 h at 37 °C in 5% CO_2_. The adhesion assay, which quantifies total cell-associated bacteria (surface-adherent and intracellular bacteria), and invasion assay (using the antibiotic protection assay) were both performed as previously described [34].

### Whole blood bactericidal (killing) assay

Blood was collected from 6 to 10 week-old CD1 mice (Charles River Laboratories, Wilmington, MA) and mixed with sodium heparin (Sigma-Aldrich). The test was performed as previously described, with a few modifications [38]. Briefly, leukocytes (9 × 10^6^ cells/mL on average) were transferred to a microtube containing around 1 × 10^7^ CFU/mL of the different *S. suis* strains (MOI = 1) and incubated for 2 h, mixing every 20 min. After incubation, cells were lysed, and appropriate dilutions plated on THA to determine viable bacterial counts. Resistance to bacterial killing by blood leukocytes was compared to incubation in plasma alone (obtained by centrifuging whole blood at 1800 × *g* for 10 min at 4 °C). The percentage of bacteria killed was determined using the following formula: 1 − (bacteria in blood/bacteria in plasma) × 100%.

### *S. suis* activation of marrow-derived dendritic cells (bmDC)

The femur and tibia from CD1 mice (Charles River Laboratories) were used to generate bmDCs, as previously described [39]. Briefly, hematopoietic bone marrow stem cells were cultured in complete culture medium (RPMI-1640 supplemented with 5% heat-inactivated fetal bovine serum, 10 mM HEPES, 2 mM l-glutamine, and 50 µM 2-mercaptoethanol (Gibco, Burlington, ON, Canada) and complemented with 20% granulocyte-macrophages colony-stimulating factor from mouse-transfected Ag8653 cells [40]. Cell purity was confirmed to be at least 90% CD11c + (a marker for DCs) by flow cytometry as previously described [39]. Albeit this culture system cannot completely rule out the presence of other innate cells such as macrophages, it represents an enriched source of bmDCs.

All experiments were performed in the absence of endotoxin (lipopolysaccharide) contamination and under non-toxic conditions (data not shown), the latter being evaluated by the lactate dehydrogenase release with the CytoTox 96^®^ Non-Radioactive Cytotoxicity Assay (Promega, Madison, WI, USA). Prior to infection, cells were resuspended at 1 × 10^6^ cells/mL in complete medium and stimulated with the different live *S. suis* strains (1 × 10^6^ CFU/mL; initial MOI = 1). Conditions used were based on those previously published [5, 24]. Supernatants were collected at different incubation times (4, 6, 8 and 12 h following infection with *S. suis*), to measure cytokine release under non-toxic conditions (not shown). Mock-infected cells served as negative controls. Secreted levels of pro-inflammatory cytokines produced during the acute phase of inflammation caused by *S. suis*, namely: tumor necrosis factor (TNF), interleukin (IL)-6, C–C motif chemokine ligand (CCL) 3, and C–X–C motif chemokine ligand (CXCL) 1 were quantified by sandwich ELISA using pair-matched antibodies from R&D Systems (Minneapolis, MN, USA) according to the manufacturer’s recommendations. Among others, these pro-inflammatory cytokines have been previously been shown to be induced by S*. suis* [7, 21, 24].

### *S. suis* virulence mouse model of systemic infection

A C57BL/6J mouse model of infection was used [6]. As mentioned above, these studies were carried out in strict accordance with the recommendations of and approved by the University of Montreal Animal Welfare Committee guidelines and policies, including euthanasia to minimize animal suffering by the use of humane endpoints, applied throughout this study when animals were seriously affected (mortality was not an endpoint measurement). Thirty six week-old female C57BL/6J (Jackson Research Laboratories, Bar Harbor, ME, USA) were used for these experiments (15 mice per group). Early stationary phase bacteria were washed twice in phosphate-buffered saline, pH 7.4, and resuspended in THB [38, 41]. Bacterial cultures were appropriately diluted and plated on THB agar (THA) to accurately determine bacterial concentrations. Mice were inoculated with 1 × 10^7^ CFU via the intraperitoneal route and health and behavior monitored at least thrice daily until 72 h post-infection and twice thereafter until the end of the experiment (12 days post-infection) for the development of clinical signs of sepsis, such as depression, swollen eyes, rough hair coat, prostration, and lethargy. For bacteremia studies, blood samples were collected from the caudal vein of surviving mice 12 h, 24 h, and 48 h post-infection and plated as previously described [6].

### Statistical analyses

Normality of data was verified using the Shapiro–Wilk test. Accordingly, parametric (unpaired t-test) or non-parametric tests (Mann–Whitney rank sum test), where appropriate, were performed to evaluate statistical differences between groups. Log-rank test was used to compare survival rates between wild-type-infected mice and those infected with mutant strains. Each in vitro test was repeated in at least three independent experiments. *p* < 0.05 was considered as statistically significant.

## Results

### LtaS is required for the LTA synthesis in *S. suis*

In other Gram-positive bacteria, the LtaS enzyme mediates the polymerization of the poly-glycerolphosphate chain by the repeated addition of GroP residues to the tip of the growing chain using the lipid phosphatidylglycerol (PG) as substrate [12, 42–45]. We targeted for mutagenesis a gene (SSU_RS05590*, ltaS*) encoding this enzyme in *S. suis* strain P1/7 [46]. After confirmation of the *ltaS* deletion, we performed a large-scale extraction of LTA from the P1/7 wild-type and the Δ*ltaS* mutant strains following our established protocol [7]. LTA-containing fractions are indicated by a positive inorganic phosphate test (mainly pool 2 in the wild-type, Figure 1A top panel). Such phosphate positive fractions were absent in runs from extractions of the Δ*ltaS* mutant strain (Figure 1A bottom panel). This absence of phosphate positive fractions in the typical LTA-fraction range is a clear indication that the mutant samples are devoid of LTA. For further corroboration, pooled fractions were subjected to native Tris-tricine-PAGE analysis with subsequent alcian blue and silver staining (Figure 1B) following a published protocol [29, 30]. This proved the presence of LTA mainly in pool 2, but partially also in pool 1 of the HIC purification of LTA isolated from *S. suis* P1/7 wild-type. No LTA representing band pattern could be detected in the extracts of the Δ*ltaS* mutant. Taken together, these results unequivocally indicate that the enzyme encoded by the *LtaS* gene is essential for the production of LTA in *S. suis*.

### Absence of LTA does not lead to an increase in hydrophobicity of the bacterial surface

Along with peptidoglycan and lipoproteins, LTA are major components of the cell wall of Gram-positive bacteria [6]. Considering LTA’s crucial location, its absence might affect the presence or distribution of the capsular polysaccharide (CPS) at the bacterial surface, thereby affecting the hydrophobicity of the mutant strain. As anticipated, an unencapsulated *cpsF* mutant (used as a positive control) presented an increased surface hydrophobicity (positive control, Additional file 1). However, no significant differences in hydrophobicity were observed between the wild-type and the Δ*ltaS* mutant strains (Additional file 1). This indicates that LTA does not significantly influence surface hydrophobicity, possibly due to the overriding presence of CPS.

### Absence of LTA does not affect *S. suis* growth in rich and poor media

In *S. aureus,* LtaS is required for efficient cell growth [12, 47, 48], whereas a Δ*ltaS* mutant of *Streptococcus gordonii* displayed just a delayed growth [49]*.* Thus, to investigate the role of LTA in the growth of *S. suis*, we grew both wild-type and Δ*ltaS* mutant strains in two different media (Additional file 2). When cultured in rich media (THB), both strains exhibited similar, robust growth rates as assessed by bacterial counts (Additional file 2A). Similarly, when grown in plasma (a less nutrient-rich medium that mimics in vivo conditions), the growth of both strains was comparable (Additional file 2B). Even if growth of the Δ*ltaS* mutant is similar (in CFU) to that of the wild-type strain, the suspension of the Δ*ltaS* mutant appeared to be filamentous in comparison to a homogenous growth of the wild-type (Additional file 3). Additionally, homogenizing the bacterial suspensions of the mutant was challenging. This phenotype has been observed in other Gram-positive bacteria [50–52].

### Absence of LTA leads to increased self-aggregation and biofilm formation

**Figure 2:**
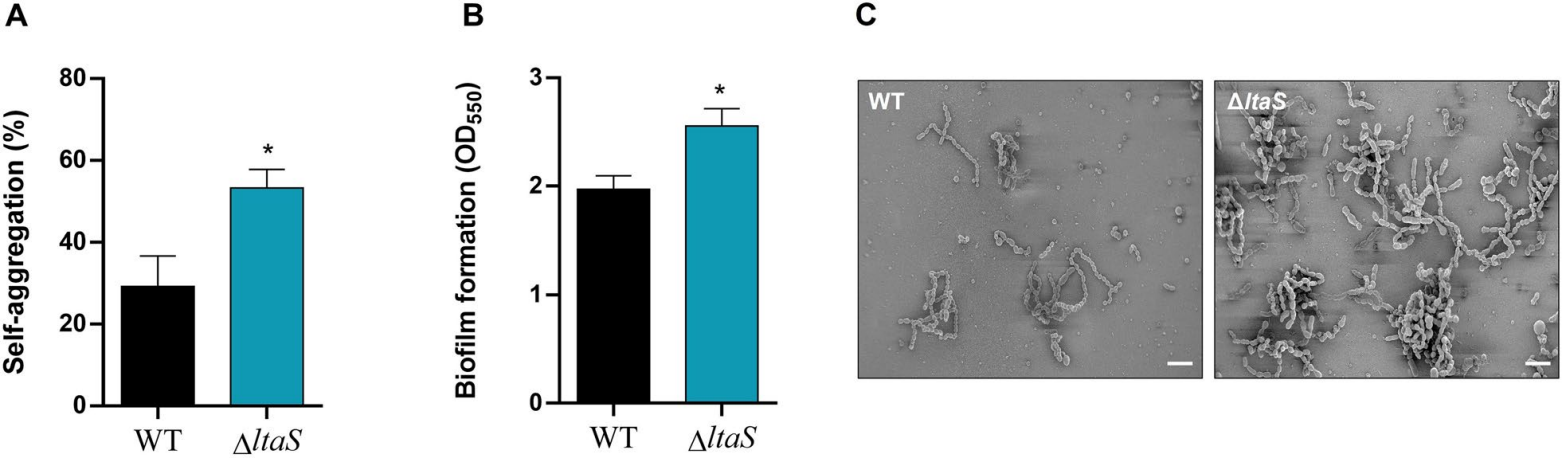
**LtaS mutation leads to increased self-aggregation and biofilm formation.** Cell-to-cell aggregation in fluid phase (**A**) and biofilm formation capacity in the presence of porcine fibrinogen (**B**) after 24 h of incubation at 37 °C of *S. suis* wild-type P1/7 strain (black) and Δ*ltaS* (blue). Data represent the mean ± SEM from at least three independent experiments. *(*p* < 0.05) indicate a significant difference between the wild-type and Δ*ltaS* mutant. (**C**) Scanning electron micrographs (SEM) of wild-type P1/7 strain (black) and Δ*ltaS* (blue), × 2.00 k (scale bar: 40.0 µm). The micrographs are representative of the microscopic fields observed on the slide.

Due to the observations mentioned above, we conducted self-aggregation and biofilm assays (Figures 2A and B). Deletion of LtaS significantly increased self-aggregation of *S. suis* by 25% (Figure 2A). Furthermore, the Δ*ltaS* mutant strain showed a significantly greater capacity to form biofilm than the wild-type strain in the presence of porcine fibrinogen (Figure 2B). Moreover, SEM micrographs showed that the Δ*ltaS* mutant tended to form more bacterial agglomerations than the wild-type strain, making it difficult to find fields with isolated bacterial chains in the mutant preparations (Figure 2C).

### LTA depletion leads to atypical bacterial morphology

**Figure 3:**
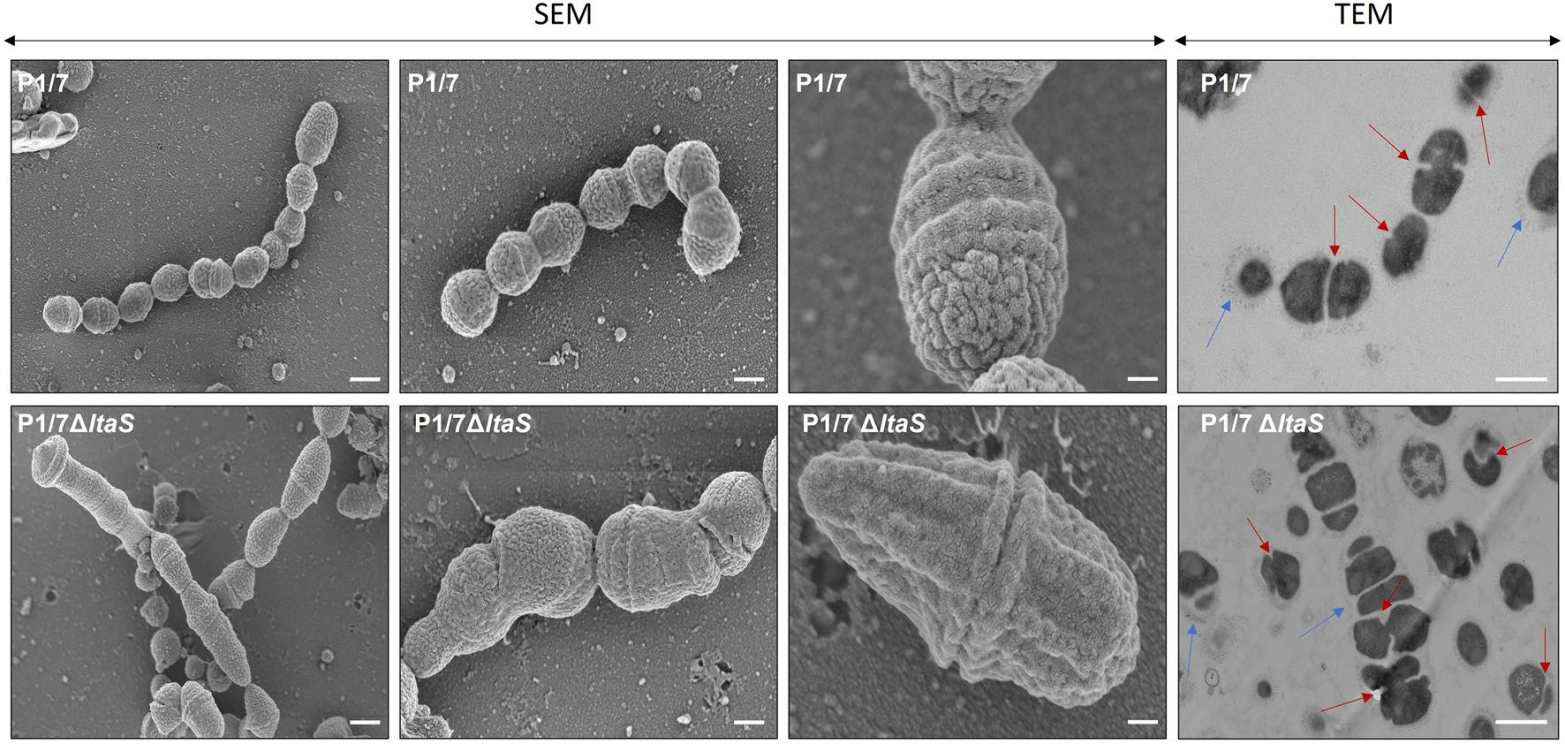
**Absence of LTA leads to cell division defects.** Scanning (SEM) and transmission (TEM) electron micrographs of the wild-type P1/7 strain and Δ*ltaS* mutant strain. Representative scanning and transmission electron micrographs of P1/7 wild-type and Δ*ltaS* mutant strains, taken at × 15.0 k (scale bar: 6.0 µm), × 25.0 k (scale bar: 4.0 µm), and × 80.0 k (scale bar: 1.0 µm), magnification for SEM and × 17.0 k (scale bar: 0.5 µm) for TEM. Red arrows ( →) denote septa and blue arrows ( →) indicate the presence of capsular polysaccharide.

LTA plays an important role in bacterial physiology and it contributes to membrane homeostasis, as evidenced in *S. aureus* [48]. Here, SEM micrographs of the *S. suis* Δ*ltaS* mutant show defective separation of the bacterial cells which in addition struggle to maintain a proper coccus shape. In contrast, the wild-type strain displays regularly sized coccus-shaped cells (Figure 3). TEM micrographs suggest that this division defect might stem from irregular septa placement during cell division. In the Δ*ltaS* mutant, septa placement appears erratic, whereas in the wild-type, septa are consistently aligned in the same plane (Figure 3).

### *S. suis* LTA contributes to cationic antimicrobial peptide (CAMPs) resistance

The minimal inhibitory concentrations (MICs) of membrane-damaging antimicrobial peptides colistin, polymyxin B and nisin were determined for the wild-type P1/7 and the Δ*ltaS* mutant strains. The MIC of nisin was the same for both strains (Table 3). However, cationic peptides colistin and polymyxin B, the MICs were significantly higher in the wild-type strain compared to the Δ*ltaS* mutant (Table 3). These results suggest that LTA play a role in the intrinsic resistance of *S. suis* to the destruction by certain CAMPs.

**Table 3:** **Sensitivity of the** ***S. suis*** ***wild-type P1/7 and ΔltaS* mutant strains to the action of selected antimicrobial peptides.**

**Strains**	**MICs (μg/mL)**		
	**Colistin**	**Polymixin B**	**Nisin**
Wild-type	200	75	40
Δ*ltaS*	50	37.5	40

### Lack of LTA does not impair the adhesion to and invasion of swine epithelial cells

**Figure 4:**
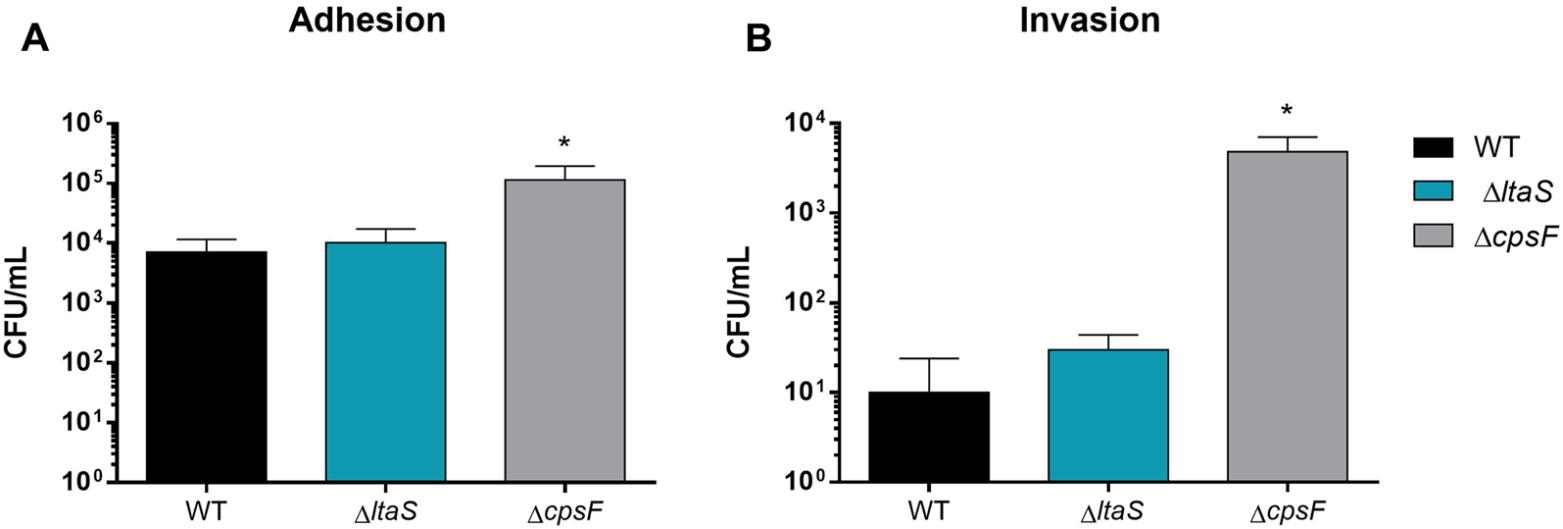
**The Δ*ltaS* mutant is not impaired in its capacity of adhesion to and invasion of epithelial cells.** Adhesion (**A**) and invasion (**B**) to NPTr of the *S. suis* wild-type P1/7 strain (black), Δ*ltaS* (blue) and Δ*cpsF* (grey) mutant strains after 2 h of incubation. *Indicates a significant difference (*p* < 0.05). Each bar represents the mean bacterial concentration (CFU/mL) + SEM from at least three independent experiments.

LTA has been proposed to mediate initial interactions with human epithelial cells and previous studies using *ΔdltA* mutants in diverse bacterial species have revealed a direct role of D-alanylation of LTA in regulating adhesion to and invasion of host cells. Thus, we evaluated the role of LTA in the *S. suis* adhesion/invasion to NPTr cells using the Δ*ltaS* mutant strain. As anticipated, the non-encapsulated mutant strain significantly adhered and invaded cells more efficiently than the wild-type strain (positive control, Figure 4). However, the adhesion and invasion rates of the Δ*ltaS* mutant were similar to those of the wild-type strain (Figure 4). These results indicate that the LTA does not play a significant role in in *S. suis* adhesion or invasion of the epithelial cells tested.

### Presence of LTA is not required for *S. suis* in-vitro resistance to bacterial killing

**Figure 5:**
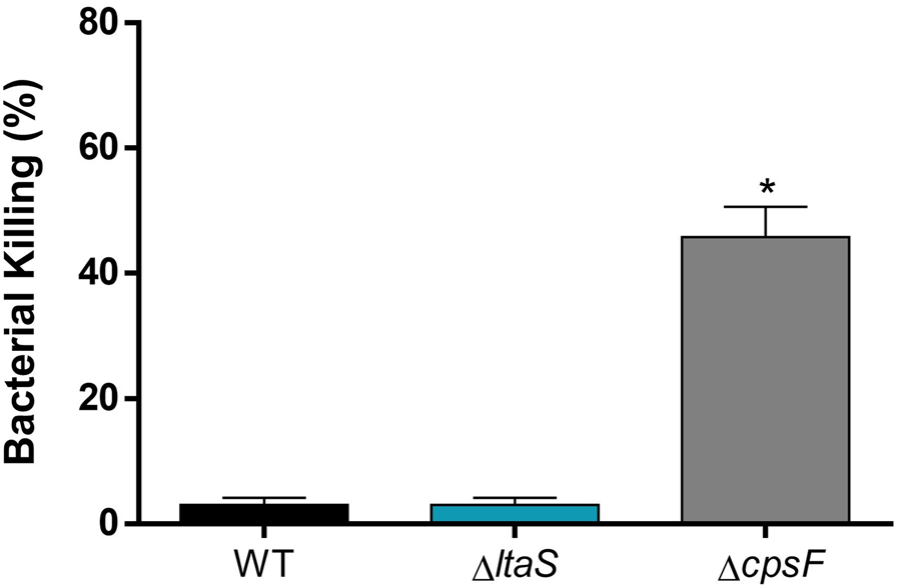
**The Δ*ltaS* mutant is not impaired in its capacity to resist blood bactericidal killing.** Capacity of the *S. suis* wild-type strain P1/7 (black), Δ*ltaS* (blue) and Δ*cpsF* (grey) mutant strains to resist the bactericidal effect of murine whole blood after 2 h of incubation. Percentage of bacterial survival was calculated in comparison to bacteria in plasma alone. Data represent the mean + SEM (*n* = 3 independent experiments). * (*p* < 0.05) indicates a significant difference between the wild-type and mutant strains.

Resistance to the bactericidal effect of blood, particularly to the killing by leukocytes, is crucial for persistence and systemic dissemination of *S*. *suis* [53]. We thus next assessed the resistance of both the wild-type and Δ*ltaS* *S*. *suis* strain to killing by mouse whole blood. Both strains exhibited almost complete resistance, with only about 1% bacterial killing observed (Figure 5). In contrast, the unencapsulated Δ*cpsF* mutant was significantly less resistant to killing, with 50% of bacterial killing (positive control, Figure 5). These results suggest that LTA is not critical for *S*. *suis* resistance to the bactericidal effect of blood.

### Lack of LTA lead to a slight increase of pro-inflammatory cytokines by innate immune cells

**Figure 6:**
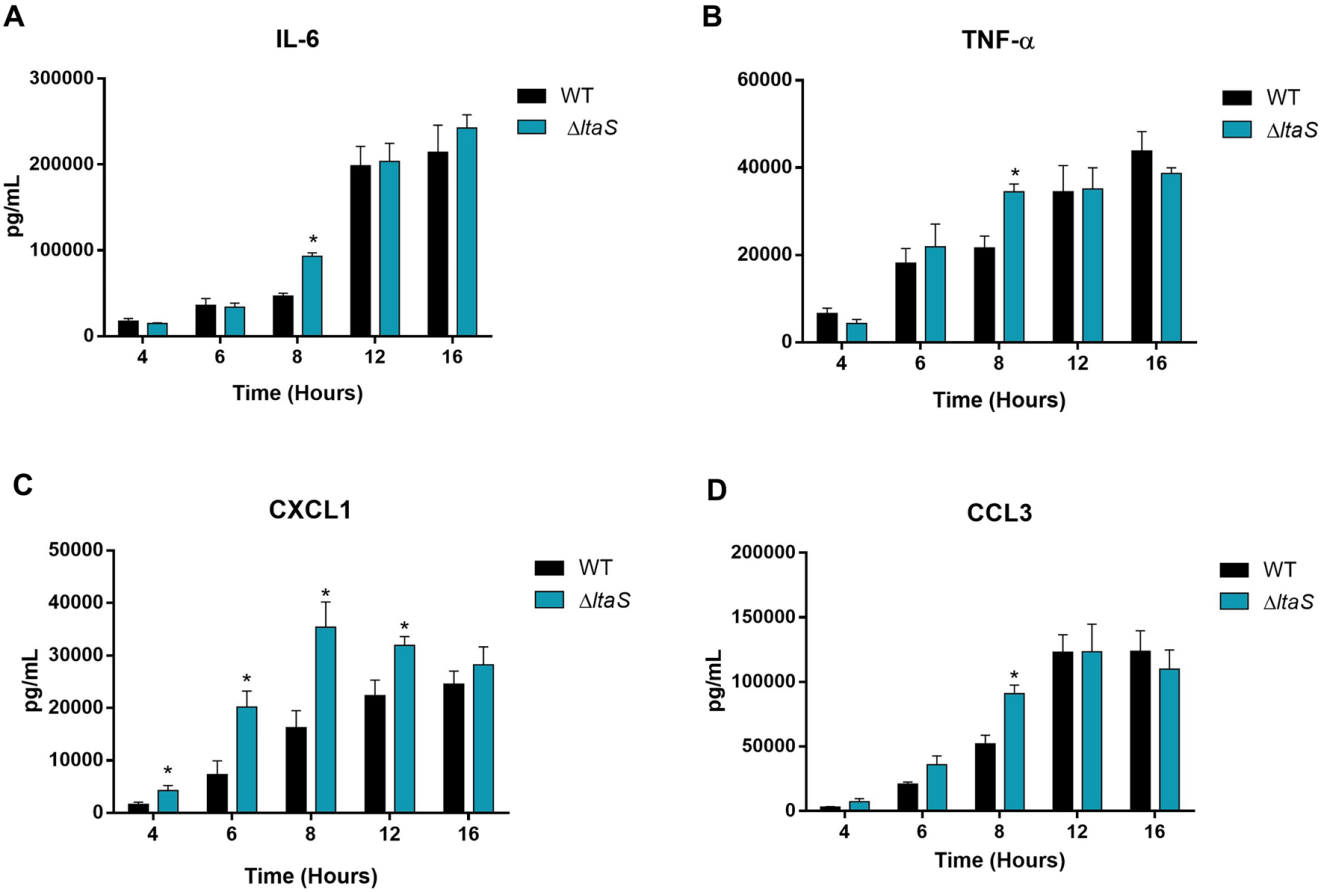
**Absence of LTA leads to a slight increase of the cytokine levels.** Pro-inflammatory cytokine production by bmDCs following activation with wild-type P1/7 strain (black) and Δ*ltaS* (blue) mutant strains. Production of IL-6 (**A**), TNF-α (**B**), CXCL1 (**C**), and CCL3 (**D**). Data represent the mean + SEM of at least three independent experiments. *(*p* < 0.05) indicates a significant difference between the wild-type and mutant strains.

DCs were used as an innate immune cell model given that these cells play a critical role during *S. suis* pathogenesis and that their inflammatory response to *S*. *suis* has been well-characterized. BmDCs were activated with bacteria for up to 16 h. In all experiments and at every incubation time, control mock infected cells showed negligible cytokine values of < 300 pg/mL (not shown). Compared to the wild-type strain, the Δ*ltaS* mutant induced significant higher levels of cytokine production only at 8 h for IL-6, TNF-α, and CCL3 (Figures 6A, B, D). For CXCL1, the increase was more pronounced with the Δ*ltaS* mutant inducing a higher CXCL1 levels from 4 to 12 h of infection (Figure 6C). However, after 12 h of incubation, levels were similar to the wild-type strain for all cytokines. These results indicate that LTA plays a limited role as *S. suis* component for pro-inflammatory cytokine release.

### Absence of LTA is not critical for *S. suis* serotype 2 systemic virulence

**Figure 7:**
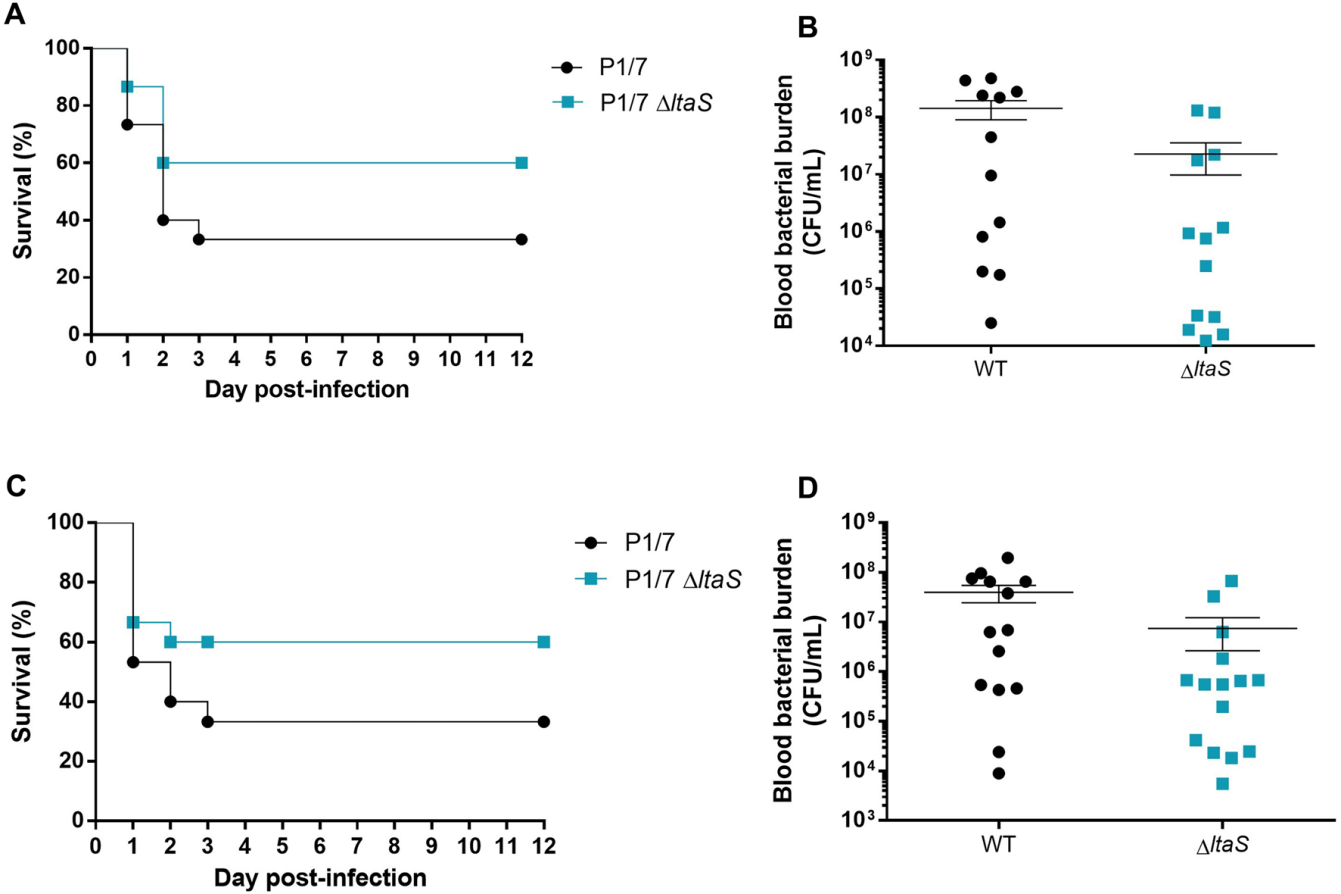
**Presence of LTA is not critical for *S. suis* systemic virulence and blood persistence following intraperitoneal inoculation.** Survival (**A**, **C**) and blood bacterial burden at 12 h post-infection (**B**, **D**) of C57BL/6 mice following intraperitoneal inoculation of the *S. suis* wild-type P1/7 strain (black) and Δ*ltaS* mutant strain (blue). Data represent survival curves (**A**, **C**) (*n* = 15) or geometric mean (**B**, **D**) (*n* = survived mice at each time point).

To evaluate the role of LTA on the systemic virulence of *S. suis*, we used a well-characterized C57BL/6 mouse model of infection. Although the absence of LTA slightly reduced *S. suis* virulence, the survival rate of mice was not significantly different from those infected with the wild-type strain (Figure 7A). Additionally, we monitored the bacterial burden in blood at early infection stages, 12, 24, and 48 h post-infection (pi). At 12 h pi, there was a marginally lower bacterial level in mice infected with the Δ*ltaS* mutant compared to the wild-type, but this difference was not statistically significant (Figure 7B). No differences were observed in bacterial counts at 24 and 48 h pi (not shown). The consistency of these findings was confirmed by repeating the experiment, yielding similar results (Figures 7C and D). Collectively, these findings indicate that LTA absence does not critically affect the systemic virulence of *S. suis* in this mouse model.

## Discussion

LTA has long been viewed as an important component of the Gram-positive cell envelope [6]. Indeed, a possible contribution of LTA to both virulence and pathogenesis of bacterial infections has been proposed. For example, it is believed to play an important role in bacterial growth and physiology, and it could contribute to membrane homeostasis [6]. In the specific case of *S. suis*, the role of LTA in virulence remained largely unknown as yet.

The polyglycerolphosphate chain of type I LTA is synthesized by LtaS, an enzyme crucial for LTA synthesis [6]. Indeed, in other bacterial species, a mutant lacking the LtaS enzyme showed a complete absence of LTA. In the present study, we demonstrate that a *S. suis* Δ*ltaS* mutant also lacks complete expression of LTA [12, 50, 52, 54]. While a functional LtaS is required for bacterial growth in *S. aureus*, *Bacillus anthracis*, *B*. *subtilis*, and *L. monocytogenes*, this does not appear to be the case for *S. suis*, as the Δ*ltaS* mutant grew comparably to the wild-type parental strain in both rich and poor media. Our findings agree with those obtained in other streptococci such as *S*. *gordonii* [55]. Absence of LTA due to TacL-deletion in *S. pneumoniae* does not influence proper growth as well [56]. However, this is not directly comparable to the situation in *S. suis* or the other mentioned species, since in *S. pneumoniae* the polymeric chains of LTA and wall teichoic acids (WTA) are of same structure [56, 57] and share a common biosynthesis pathway [58], what is different in the other species [59]. However, while our study provides valuable insights, we acknowledge certain limitations. Our inability to generate a complemented mutant warrants cautious interpretation of our findings, as it limits the confirmation of whether observed phenotypes are solely due to the targeted genetic alteration. Nonetheless, the consistency of our results with existing literature on LTA-negative mutants in other bacterial species supports the validity of our observations [49–52, 56].

Despite the lack of LTA having no impact on *S. suis* growth, the Δ*ltaS* mutant exhibited a defect in separation and bacterial cells did not have a typical coccus shape. It has been suggested that LTA may play an important role in bacterial division and separation for some bacterial species but not for others [47, 60]. Moreover, certain bacteria-bacteria interactions were altered in the absence of LTA in *S. suis*. Notably, the Δ*ltaS* mutant exhibited higher levels of self-aggregation compared to the wild-type strain. This could be due to changes in surface protein expression, such as the antigen I/II protein, involved in self-aggregation [36]. Similarly, the absence of LTA increases the capacity of *S. suis* to form biofilm. It is known that an LTA deficit can reduce the cell surface's negative charge, influencing biofilm formation [61]. In contrast to our findings, biofilm formation in the absence of LTA was decreased in species like *S. aureus*, *S. gordonii*, and *Enterococcus faecalis* [55, 62, 63]. LTA has also been shown to contribute to late-stage biofilm development for *Streptococcus mutans* through interactions with extracellular DNA. However, this mechanism does not appear to apply to *S. suis*. As observed with self-aggregation, enhanced exposure of biofilm-forming proteins in the absence of LTA in *S. suis* is a possibility [36, 64]. Additionally, *S. gordonii* LTA has been demonstrated to play a significant role in surface protein presentation, including those involved in biofilm formation. Remarkably, no modification on the surface hydrophobicity was noted in the absence of LTA. Different from our results, LTA was shown to play an important role in surface properties and hydrophobicity in *S. aureus* and *Streptococcus pyogenes* [63, 65]. In *S. suis*, the low hydrophobicity levels observed with the Δ*ltaS* mutant also suggest that the presence of the capsule was intact. Indeed, it has been previously shown that *S. suis* stains lacking capsular material present high hydrophobicity levels [66]. In addition, the capsule could be observed in the electron-microscopy pictures (Fig. 3). Finally the Δ*ltaS* mutant presented a clear positive reaction as a serotype 2 by the coagglutination test (results not shown), confirming the presence of the capsule.

LTA has long been compared to the LPS of Gram-negative bacteria [18], which is a component recognized by immune cells and participates in the initiation of the inflammatory response. Although this hypothesis is still proposed in recent publications [67, 68], other studies have challenged this role for the LTA [21, 60] and it is now accepted that lipoproteins (and not LTA) are the main activators of the innate immune system [7, 20, 21, 69]. To further study the role of LTA in inflammation caused by *S. suis*, activation of DCs by the mutant lacking LTA was compared to the wild-type strain. As expected, we confirmed that the presence of LTA is not critical for the release of cytokines by immune cells, since its absence did not lower the level of cytokines produced by DCs. On the contrary, LTA absence slightly increased the production level of cytokines. The same phenotype was observed in *S. gordonii.* Indeed, a Δ*ltaS* mutant of the latter bacterial species induced more pro-inflammatory cytokines in DCs and macrophages than the wild-type strain [60]. As proposed for *S. gordonii*, it may be also hypothesized that, in the absence of LTA, lipoproteins may be better exposed or encounter the TLR2 receptor more frequently, since LTA can bind to TLR2 but are not capable of inducing pro-inflammatory signaling through TLR1/TLR2- or TLR2/TLR6-heterodimer formation as lipoproteins do [70]. It has also been recently suggested that LTA is not a major immuno-stimulating agent but rather it interferes with bacteria-induced DC maturation in *S. gordonii* [49].

Previous studies indicated that LTA d-alanylation contributes to resistance to CAMPs. In the current study, the Δ*ltaS* mutant showed a slightly increased sensitivity to polymixin B and colistin, but not to nisin. This suggests that LTA collaborates with the intrinsic resistance of *S. suis* to CAMP killing. However, distinguishing whether this susceptibility increase is due to LTA absence or to reduced d-alanine on the bacterial surface remains challenging. Indeed, the Δ*dltA* [8, 9] and Δ*ltaS* (this study) *S. suis* mutants show differences in other aspects of the pathogenesis of the infection. Despite the known importance of LTA d-alanylation in adhesion, invasion, resistance to blood killing, and virulence [8, 9], no such differences were found between the wild-type and the Δ*ltaS* mutant strain in this study. However, it should be considered that the Δ*ltaS* mutant likely retains d-alanylation motifs. This could occur if the wall teichoic acids (WTA), which are prevalent peptidoglycan-linked polymers in many Gram-positive organisms, undergo d-alanylation [59, 71]. While the presence of WTA in *S. suis* has not yet been demonstrated, analyzing a Δ*ltaS*/Δ*dltA* double mutant could shed more light on this matter. Additionally, quantifying d-alanine in the WTA pool of both the wild-type strain and the Δ*ltaS* mutant would be insightful. Unfortunately, reliable analytical tools are yet missing for addressing this question.

In terms of *S. suis* virulence, using a well-standardized mouse model, LTA does not appear to play a significant role. Although the Δ*ltaS* mutant showed a minor decrease in virulence and blood burden in comparison to the wild-type strain in two separate experimental infections, the differences were not statistically significant. These findings align with those from similar mutants in *S. gordonii* [60]. As mentioned above, different outcomes, however, were noted with a *S. suis* mutant deficient in LTA d-alanylation [6]. Indeed, in *S. suis*, LTA d-alanylation is critical for virulence in both mouse and pig models [8]. Evaluation of the Δ*ltaS* mutant by experimental infection of conventional pigs, the natural host, using a systemic infection model, is recommended for conclusive results. In addition, although no role of LTA in adhesion/invasion capacities of *S. suis* to swine epithelial cell could be observed, not all aspects of the first steps of the pathogenesis of the infection have been addressed in the current study, suggesting that further research on the LTA role(s) is needed.

Our study suggests that while LTA is involved in maintaining *S. suis* bacterial fitness and a proper cell shape, within the conditions tested, and taking into account the limitations mentioned in the discussion, its role in the pathogenesis of the *S. suis* infection appears limited. Indeed, LTA presence reduces self-agglutination, biofilm formation and even cell activation, which are important aspects of the pathogenesis of the infection caused by *S. suis*. In addition, it does not seem to play a critical role in virulence using the systemic mouse model of infection. Since the characteristics of the LTA produced by a *S. suis* strain can vary based on the genetic background and serotype of the strain [7], it is plausible that LTA may play differing roles in pathogenesis depending on these factors. Therefore, the specific influence of LTA in the pathogenesis of *S. suis* might not be uniform across different strains and serotypes.

### Supplementary Information

**Additional file 1. Surface hydrophobicity of the wild-type P1/7 strain (black), Δ*ltaS* (blue) and Δ*cpsF* (grey) strains was determined using *n*-hexadecane.** Data represent the mean ± SEM from at least three independent experiments. * (*p* < 0.05) indicates a significant difference between the Δ*cpsF *mutant strain with both the wild-type and Δ*ltaS* strains.

**Additional file 2. Growth of the wild-type P1/7 strain (black) and Δ*ltaS* (blue) in THB (A) and plasma (B). **Each point represents mean bacterial concentration (CFU/mL) ± SEM of at least three different independent experiments.

**Additional file 3. Visual aspect after 24 h growth in Todd-Hewitt broth of *S. suis* serotype 2 wild-type P1/7 strain (left) and Δ*ltaS* mutant strain (right).**

## Abbreviations

Ap: Ampicillin
CFU: colony-forming unit
CCL: C–C motif chemokine ligand
CXCL: C–X–C motif chemokine ligand
DC: dendritic cell
ELISA: enzyme-linked immunosorbent assay
Ig: Immunoglobulin
IL: interleukin
Km: Kanamycin
LB: Luria-Bertani
LTA: Lipoteichoic acid
Nptr: neonatal porcine tracheal epithelial cell
PCR: polymerase chain reaction
ST: sequence type
THA: THB agar
THB: Todd Hewitt Broth
TNF: tumor necrosis factor
WTA: wall teichoic acids
