# Supplementary figures and images for: Lipoteichoic acids influence cell shape and bacterial division of Streptococcus suis serotype 2, but play a limited role in the pathogenesis of the infection
Source: Vet Res. 2024 Mar 19;55:34. doi: 10.1186/s13567-024-01287-w (PMC10953176; doi:10.1186/s13567-024-01287-w)

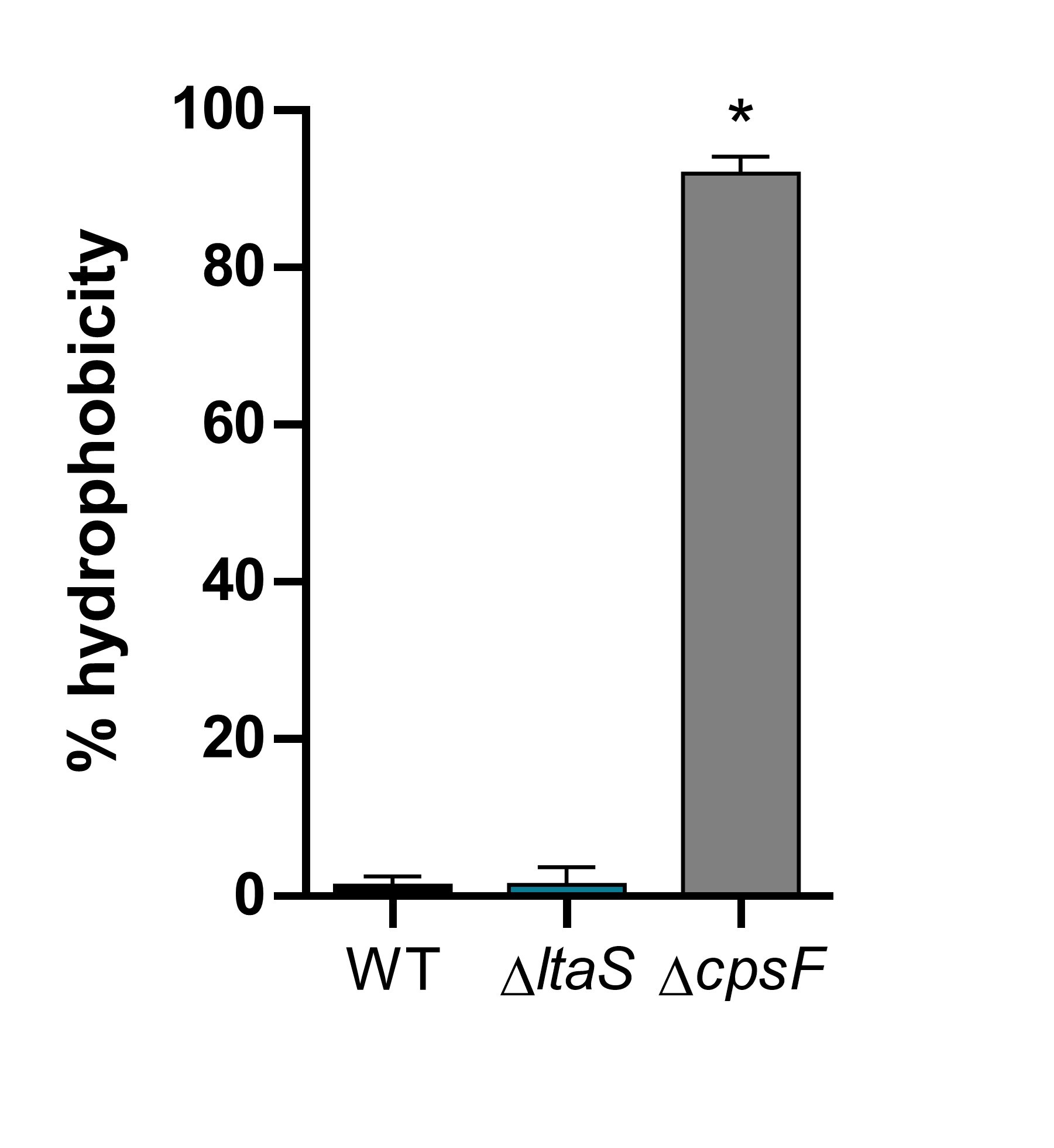

Supplement: Supplementary file 1 — Additional file 1. Surface hydrophobicity of the wild-type P1/7 strain (black), ΔltaS (blue) and ΔcpsF (grey) strains was determined using n-hexadecane. Data represent the mean ± SEM from at least three independent experiments. * (p < 0.05) indicates a significant difference between the ΔcpsF mutant strain with both the wild-type and ΔltaS strains. [file 13567_2024_1287_MOESM1_ESM.jpg]

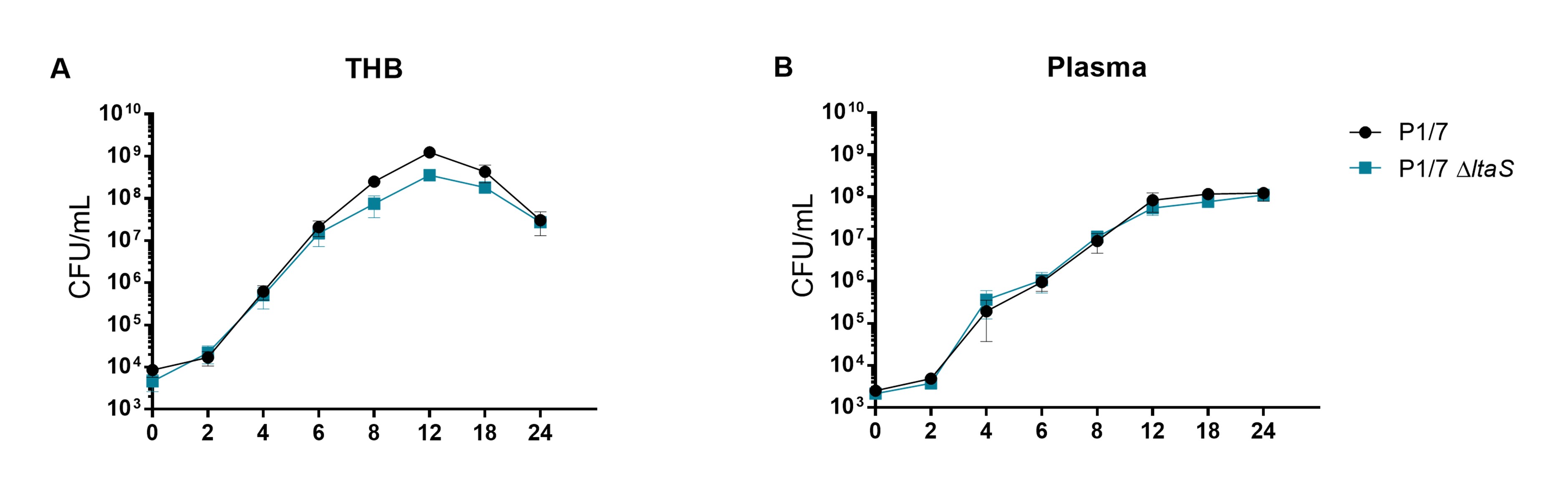

Supplement: Supplementary file 2 — Additional file 2. Growth of the wild-type P1/7 strain (black) and ΔltaS (blue) in THB (A) and plasma (B). Each point represents mean bacterial concentration (CFU/mL) ± SEM of at least three different independent experiments. [file 13567_2024_1287_MOESM2_ESM.jpg]

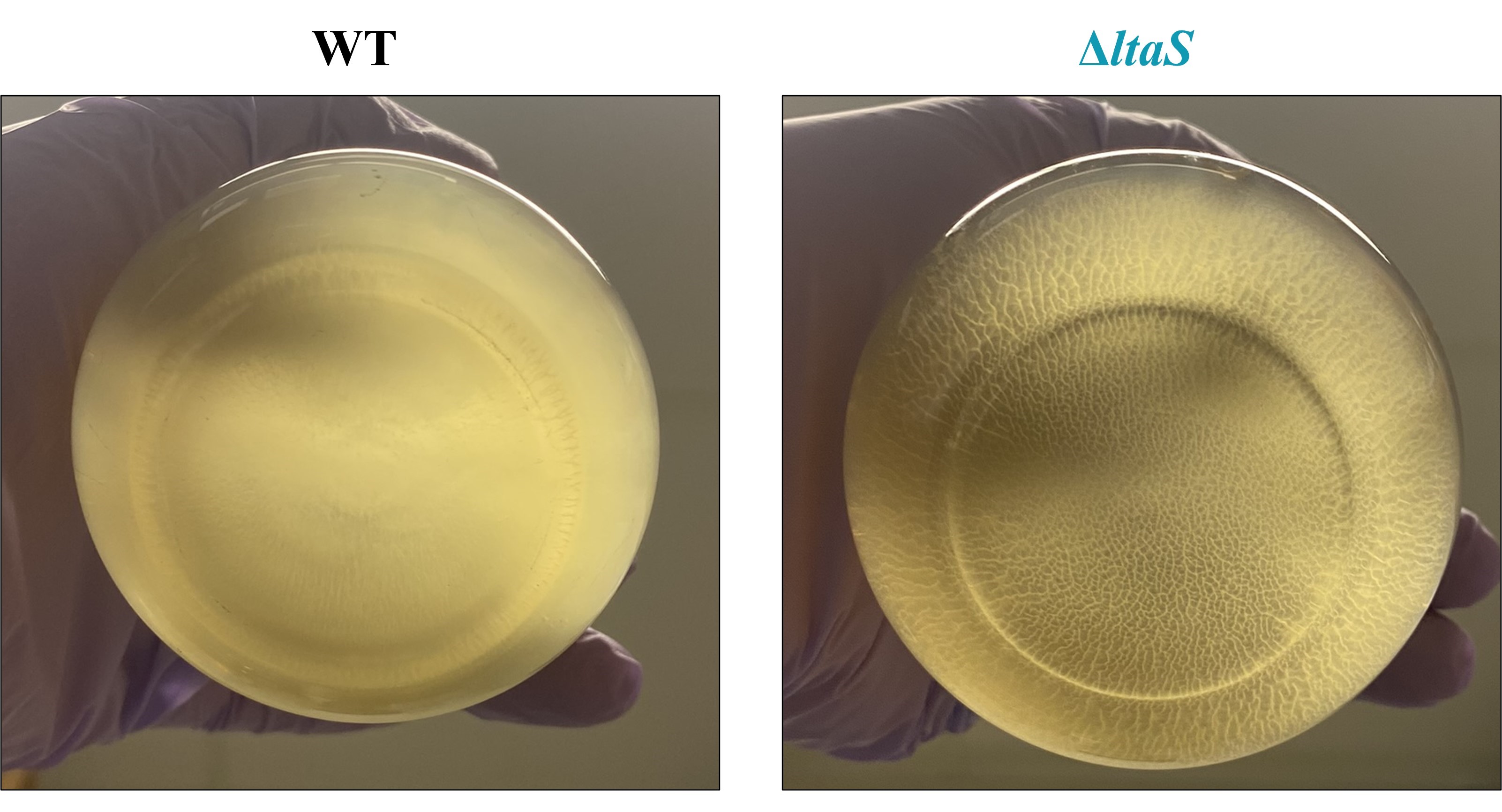

Supplement: Supplementary file 3 — Additional file 3. Visual aspect after 24 h growth in Todd-Hewitt broth of S. suis serotype 2 wild-type P1/7 strain (left) and ΔltaS mutant strain (right). [file 13567_2024_1287_MOESM3_ESM.jpg]
